# Spinal cord injury in infancy: activity-based therapy impact on health, function, and quality of life in chronic injury

**DOI:** 10.1038/s41394-020-0261-1

**Published:** 2020-03-10

**Authors:** Laura C. Argetsinger, Goutam Singh, Scott G. Bickel, Margaret L. Calvery, Andrea L. Behrman

**Affiliations:** 1 Frazier Rehab Institute, Pediatric NeuroRecovery Program, Louisville, KY USA; 2 Kosair Charities Center for Pediatric NeuroRecovery, University of Louisville, Louisville, KY USA; 3 Kentucky Spinal Cord Injury Research Center, University of Louisville, Louisville, KY USA; 4 Department of Neurological Surgery, University of Louisville, Louisville, KY USA; 5 Department of Pediatrics, University of Louisville, Louisville, KY USA

**Keywords:** Rehabilitation, Quality of life, Paediatrics

## Abstract

**Introduction:**

Spinal cord injury (SCI) in infancy magnifies the complexity of a devastating diagnosis. Children injured so young have high incidences of scoliosis, hip dysplasia, and respiratory complications leading to poor health and outcomes. We report the medical history, progression of rehabilitation, usual care and activity-based therapy, and outcomes for a child injured in infancy. Activity-based therapy (ABT) aims to activate the neuromuscular system above and below the lesion through daily, task-specific training to improve the neuromuscular capacity, and outcomes for children with acquired SCI.

**Case presentation:**

A 3-month-old infant suffered a cervical SCI from a surgical complication with resultant tetraplegia. Until age 3, her medical complications included scoliosis, kyphosis, and pneumonia. Even with extensive physical and occupational therapy, she was fully dependent on caregivers for mobility and unable to roll, come to sit, sit, stand or walk. She initiated ABT at ~3 years old, participating for 8 months. The child’s overall neuromuscular capacity improved significantly, especially for head and trunk control, contributing to major advances in respiratory health, novel engagement with her environment, and improved physical abilities.

**Discussion:**

From injury during infancy until 3 years old, this child’s health, abilities, and complications were consistent with the predicted path of early-onset SCI. Due to her age at injury, severity and chronicity of injury, she demonstrated unexpected, meaningful changes in her neuromuscular capacity during and post-ABT associated with improved health, function and quality of life for herself and her caregivers.

## Introduction

The first year of life is rich in sensorimotor experiences. A severe medical trauma, such as spinal cord injury (SCI), occurring in an infant adds medical and health complexities to an already vulnerable, yet, highly neuroplastic developing human. Paralysis dramatically alters the process of development. Paralysis and immobility following SCI, especially when injured prior to 5 years of age, places the child at greater risk for medical complications [1]. For instance, scoliosis develops in 100% of children injured before age 10 [2, 3], while hip subluxation/dislocation occurs in 93% of children injured at <10 years of age and rises to 100% occurrence in children injured at <5 years of age [3]. Furthermore, pneumonia is the most common cause of death in individuals with SCI [4] with substantial increased work of breathing by persons with tetraplegia [5, 6].

Typical physical rehabilitation focuses on maximizing function and participation while minimizing secondary complications [7, 8]. Therapists use level of injury, defined by the International Standard for Neurological Classification of Spinal Cord Injury (ISNCSCI) [9], to predict functional expectations, set goals, plan therapy sessions, and anticipate mobility/equipment needs. Functional goals are achieved predominantly via physical strategies compensating for weakness and/or paralysis of trunk, arm, and leg muscles, and using spared musculature above the lesion. Equipment, furthermore, provides support to achieve upright sitting (e.g., thoracolumbosacral orthosis, wheelchair, cushion, chest strap), standing (e.g., static or mobile stander), and ambulation when appropriate (e.g., knee–ankle–foot orthoses, reciprocating gait orthoses, walker) [10, 11]. Recommendations for treatment intensity and duration include 2–3×/week for the first year post-SCI and 1–2×/week for the next 1–2 years [8]. In this case study, we report the medical and rehabilitation history of a child injured as an infant, who was treated using activity-based therapies (ABT) at 2 years post injury. Activity-based-locomotor training (AB-LT) [12–15] aims to activate the neuromuscular system above and below the injury using a task-specific, intense practice of manually-facilitated standing and stepping to deliver sensorimotor input and improve neuromuscular capacity and function [16]. Therapeutic outcomes for this child demonstrate a meaningful change in the trajectory of health, function, and quality of life.

## Case presentation

### Medical history

**Fig. 1:**
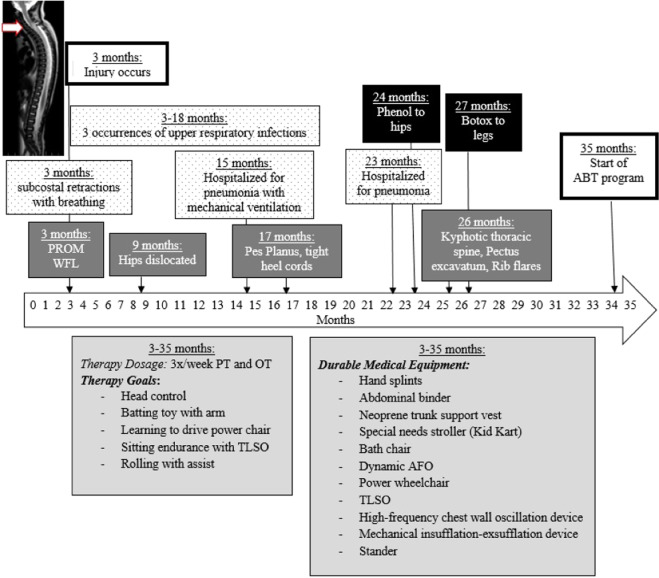
Timeline for child’s medical and therapeutic interventions and outcomes from injury onset (3 months) to initiation of activity-based therapy program (35 months). Dotted background boxes = respiratory history, dark gray boxes = musculoskeletal history, black boxes = pharmacological interventions, and light gray boxes = therapy history and equipment. PROM passive range of motion, WFL within functional limits, ABT activity-based therapy, PT physical therapy, OT occupational therapy, TLSO thoracolumbosacral orthosis, AFO ankle foot orthoses.

Medical chart review provided the history for this infant from 3 to 35 months of age. A 3-month-old female sustained a cervical SCI secondary to migration of a Broviac catheter into her spinal cord post-Ladd’s procedure for intestinal malrotation. An enlargement of the spinal cord from C5-C7 was observed (Fig. 1). Acutely, no voluntary movements of bilateral legs or distal arms were seen; lower extremity reflexes were absent. She responded to noxious stimulus only through the C6–C7 dermatomes. She remained hospitalized 3 weeks post surgery followed by 1 week of inpatient rehabilitation and then discharged home. From injury to 2 years 11 months, the medical record documents medical care addressing numerous expected health complications, e.g., respiratory, musculoskeletal, and their treatment and management (Fig. 1).

During this period, respiratory compromise was documented by three upper respiratory infections and two hospitalizations for pneumonia (one in August and one in the following March) (Fig. 1). Subcostal retractions, commonly associated with respiratory distress, also were observed during sitting. At 23 months post injury, paradoxical diaphragmatic breathing, diminished chest excursion with inspiration, poor cough efficiency, and quiet vocalizations/talking were noted. An abdominal binder was recommended for diaphragmatic support, while a high frequency chest wall oscillation vest and mechanical insufflation-exsufflation were both used to augment airway clearance abilities. From 3 to 35 months, range of motion limitations developed in her lower extremities (LEs), as well as pes planus (Fig. 1). She was diagnosed with bilateral hip dislocations (9 months) and kyphotic posture described (26 months). Pharmaceutical interventions (i.e., phenol, botox) were used addressing LE range of motion limitations. Outpatient physical (PT) and occupational therapy (OT) were attended 2×/week (45 min each), and, in-home PT (i.e., early intervention) and OT 1×/week (1 h each). Extensive medical equipment (Fig. 1) was prescribed and used with good adherence according to parents. The child received ~350 therapy sessions, including early intervention across 2.5 years.

At 2 years, 11 months of age, the child was medically approved for participation in an outpatient AB-LT program. The parent signed informed consents for approved Institutional Review Board protocols for (1) maintenance of her child’s clinical data in a secure database and for future use in dissemination of program outcomes, as well as (2) the conduct of respiratory function testing. At the time of program admission, the ISNCSCI exam was not used as it is unreliable in children <6 years of age [17]. During assessment of the child’s neurologic status, no willful movement of her LEs, wrist flexors or digits was observed. Her triceps, biceps, and wrist extensors demonstrated weakness. She utilized a weak tenodesis grasp or bilateral grasp for completion of basic tasks. A hyporesponsive reflex of the left patellar tendon was elicited otherwise LE reflexes were absent with the legs intermittently demonstrating spasms resulting in extension in supine. In sitting, the child demonstrated increased respiratory rate (35–40 breaths/min) [18]. She breathed with visible effort, elevation of upper trunk, subcostal retractions, and use of accessory muscles. Her voice was weak and quiet without projection. Her facial color appeared pale.

**Fig. 2:**
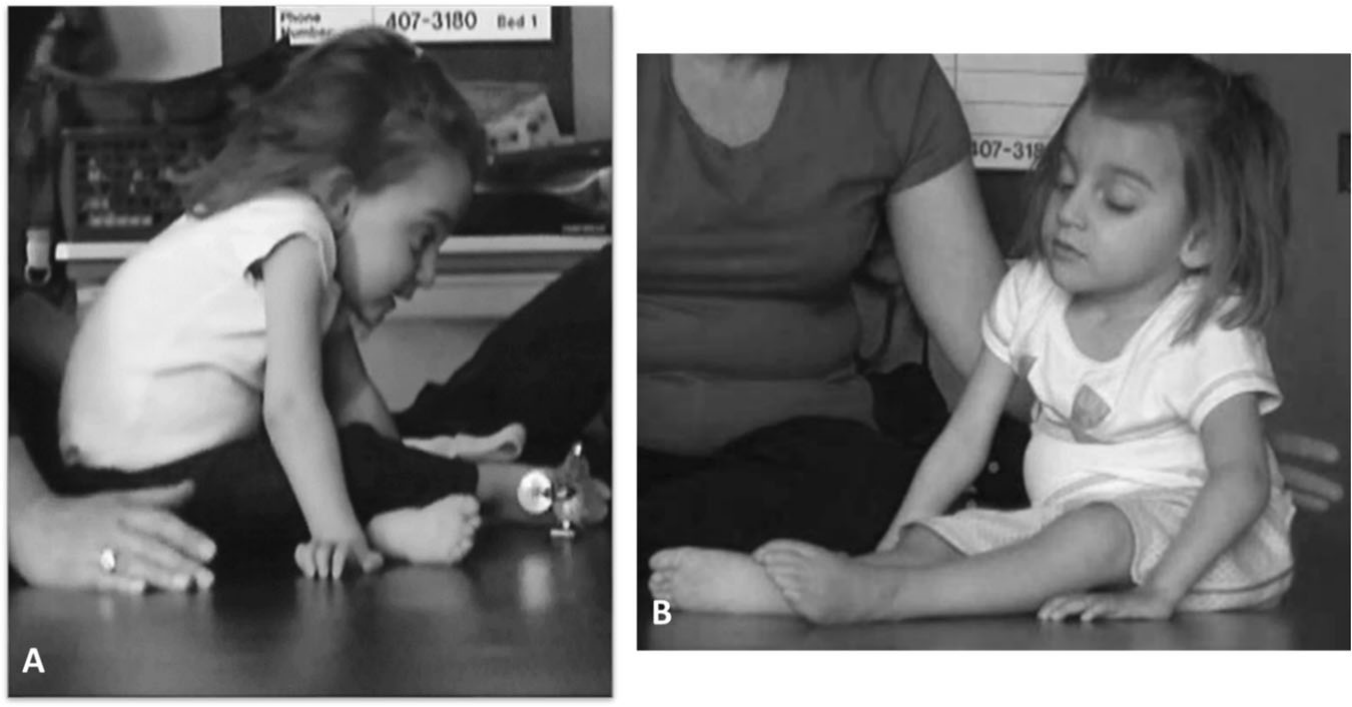
Propped ring-sitting at initial evaluation. Child was able to maintain this position for 30 s. She exhibited kyphotic spine, sacral sitting with posterior tilted pelvis (**a**), and tendency to laterally weight shift over right hip (**b**).

**Fig. 3:**
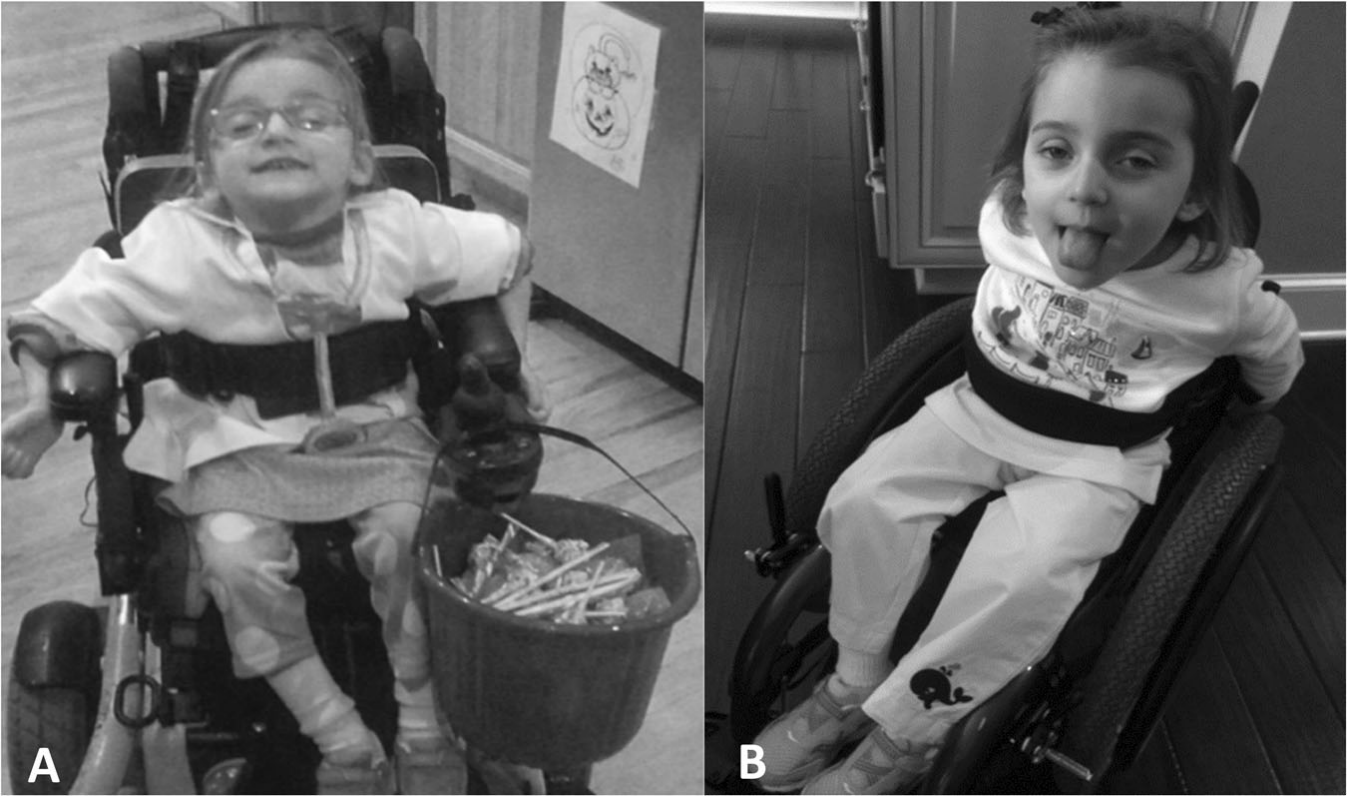
Child learning to perform independent mobility. **a** At initial evaluation, child utilized external supports and behavioral strategies to sit upright including hooking arms around armrests, chest strap, pelvic strap, and capital extension of neck to stack head over spine to position and control head. Parent report. **a** “And this is one of the things that I think people who don’t have a child with a disability take for granted as far as being able to put your child in a grocery cart. This has been a main problem for me for a long time because she couldn’t sit up. Now I can put her in a grocery cart. I don’t have to have a special seat or a special tool or a special anything. I can just go to the grocery store.” **b** By discharge, she was able to independently propel a manual wheelchair while navigating her environment using only a seat belt while maintaining trunk and head upright. Parent report. **b** “I never thought I’d have to child-proof my home with a child with a serious physical disability. She really wasn’t going any place that I didn’t take her or put her and since we’ve been here she is rolling from place to place, she’s much more physically active. And she’s getting into things that she’s not supposed to; which is good and bad, but mostly good”.

Functionally, she was unable to short-sit independently with or without arm support, requiring external support to sit upright. A timed test of sitting endurance with arm support revealed short-sitting capacity for 3 s and ring-sitting (long-sitting) for 30 s. She exhibited a kyphotic posture with posteriorly tilted pelvis (sacral sit) and lateral weight shift onto the right hip (Fig. 2a, b). The child was unable to perform independent floor or bed mobility (e.g., roll, come to sit, scoot, crawl). Her parents utilized an adaptive stroller that was reclined to support the child’s trunk and head as the primary means of transport. She was unable to propel a manual wheelchair, and power chair mobility had been initiated requiring external stabilization (i.e., reclined position with arms hooked around the armrests, a chest strap, and capital extension to stabilize her head) (Fig. 3a). She drove with her left hand over the manual control. At initiation of the ABT program, the mother described her child as “She’s not going anywhere I don’t take her or put her.”

**Fig. 4:**
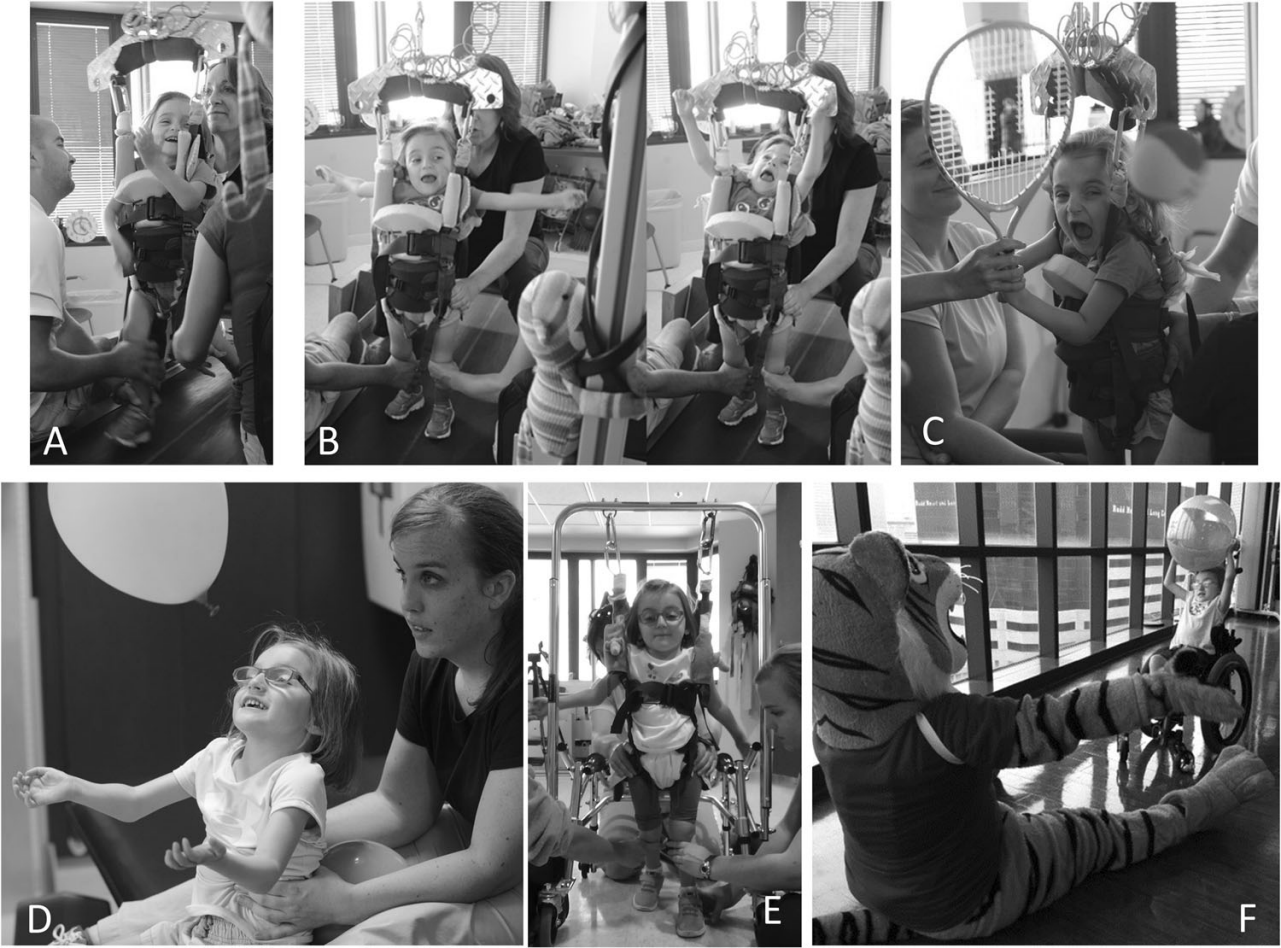
Activity-based therapy intervention. Training activities on the treadmill: **a**–**c**, and training activities off the treadmill: **d**, **e**. **a** Step retraining at age-appropriate speeds with partial body weight support and trainer facilitated stepping via manual cues. **b** Stand adaptability training activity target active trunk extension with “letter spelling” with arms to the side and overhead. **c** Stand adaptability training to facilitate trunk rotation while swinging a tennis racket and aiming at a ball. **d** Sitting with balloons passed at or above eye level to emphasize upright sitting posture and activate trunk extensors. Note therapist’s hands providing support at mid-to-low ribs and activity challenging trunk control above the support. **e** Facilitated stepping activity with manual facilitated sensory cues provided at legs and pelvis with partial body-weight support and posterior walker. **f** Community Integration. Therapists worked with family to integrate activities in the home and community to increase practice of newly developed skills by her changing neuromuscular capacity. In this instance, child is playing ball toss at a community event reinforcing overhead reach, trunk extension, and trunk control in manual wheelchair.

The child received a standardized protocol for ABT [19, 20] following AB-LT principles [19] 90 min a day 5×/week across three environments (Fig. 4). First, 1 h of neuromuscular retraining occurred in a body weight supported (BWS) environment on the treadmill with manual facilitation at the trunk, pelvis, and legs to provide sensory input for standing and stepping (Fig. 4a–c). Second, assessment and utilization of new skills followed for 30 min over ground emphasizing appropriate kinematics during activities in sitting, standing, or walking (Fig. 4d, e). Finally, therapists worked with family to identify opportunities for home and community integration to reinforce the child’s changing capacity (Fig. 4f). Clinical reassessments were performed approximately every 20 sessions.

The child received 144 of AB-LT sessions across 8 months, ~20 sessions per month. In the first 5 months, 16 cancellations occurred (medical appointments and illnesses—admitted to the local hospital for 4 days for atelectasis, and treatment of asthma, month of April). During the last 3 months, she had no cancellations. Her average stepping/treadmill velocity and endurance changed from 0.8 mph for 20 min initially to 2.0 mph for 30 min during this time period with ≤40% BWS. Therapeutic activities during standing targeted thoracic extension, rotation, and scapular retraction, e.g., overhead throw a ball/reach for toys, stretching a slinky using arm abduction, swinging a tennis racquet, and arm swing while stepping. Facilitated sitting (Fig. 4d), standing and stepping (Fig. 4e) were utilized off the treadmill to target trunk control, including head alignment/chin tuck with manually-facilitated sit-ups, reaching outside her base of support, and trunk rotation while swinging a bat. Toys handed overhead promoted active trunk extension and countered her kyphotic posture. An anterior pelvic tilt was manually assisted with segmental trunk support to achieve vertical neutral pelvic alignment during trunk and arm activities, e.g., catch and toss a balloon (Fig. 4d).

To provide integration of her therapy at home, the mother was encouraged to remove her daughter from the power chair or stroller and place the child on the floor/carpet in her home to explore and play whether in sitting or supine. The mother reported new abilities such as her daughter rolling and coming to sit independently in her bed and rolling in her sleep. In addition, a rocking chair was trialed, adapted for positioning and safety, then introduced to encourage self-initiated movement. The rocking included weight-bearing through the feet with an upright posture and though initiated first with the arms, progressed to engaging the trunk. It was hypothesized that improved trunk control and respiratory capacity would support progress and ultimately success at manual wheelchair propulsion.

**Fig. 5:**
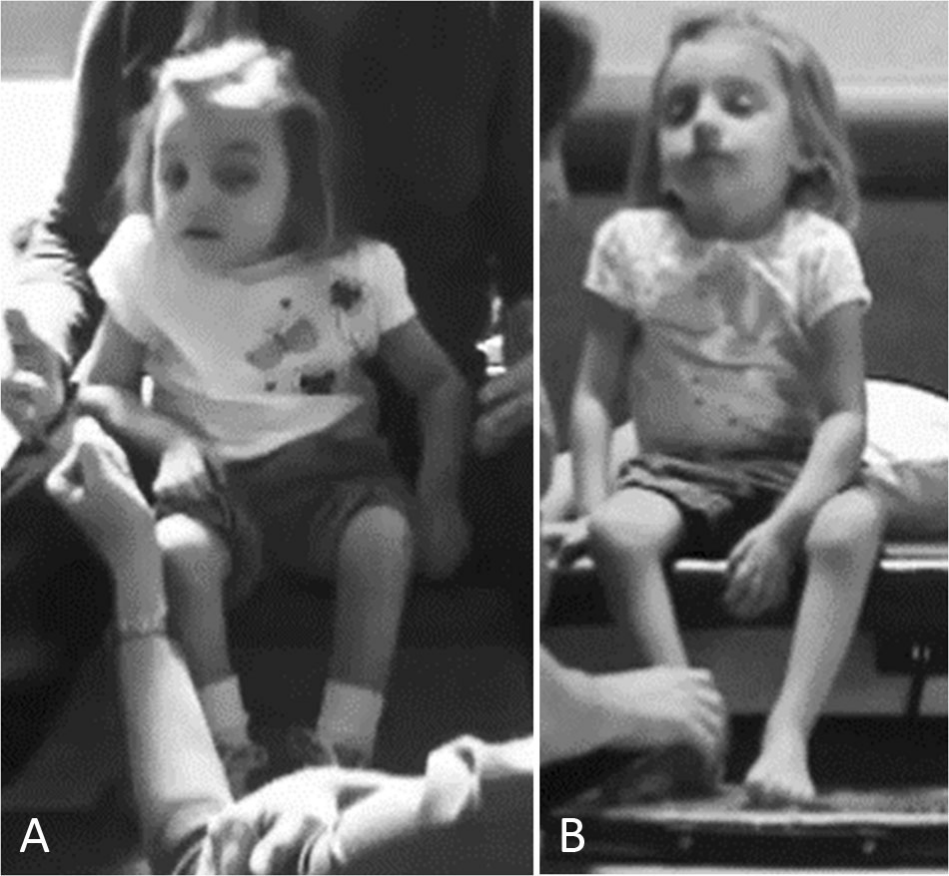
Short-sitting posture. Sitting posture at initial evaluation (**a**) and at discharge evaluation (**b**). At initial evaluation, child requires therapist assistance to sit, cannot sit independently even with arm support, and sits with flexed trunk, posterior pelvic tilt, and weight distributed to right side. **a** At discharge evaluation, child can short-sit without support by therapist and stand-by guarding only, uses minimal arm support, trunk is upright, and extended.

**Fig. 6:**
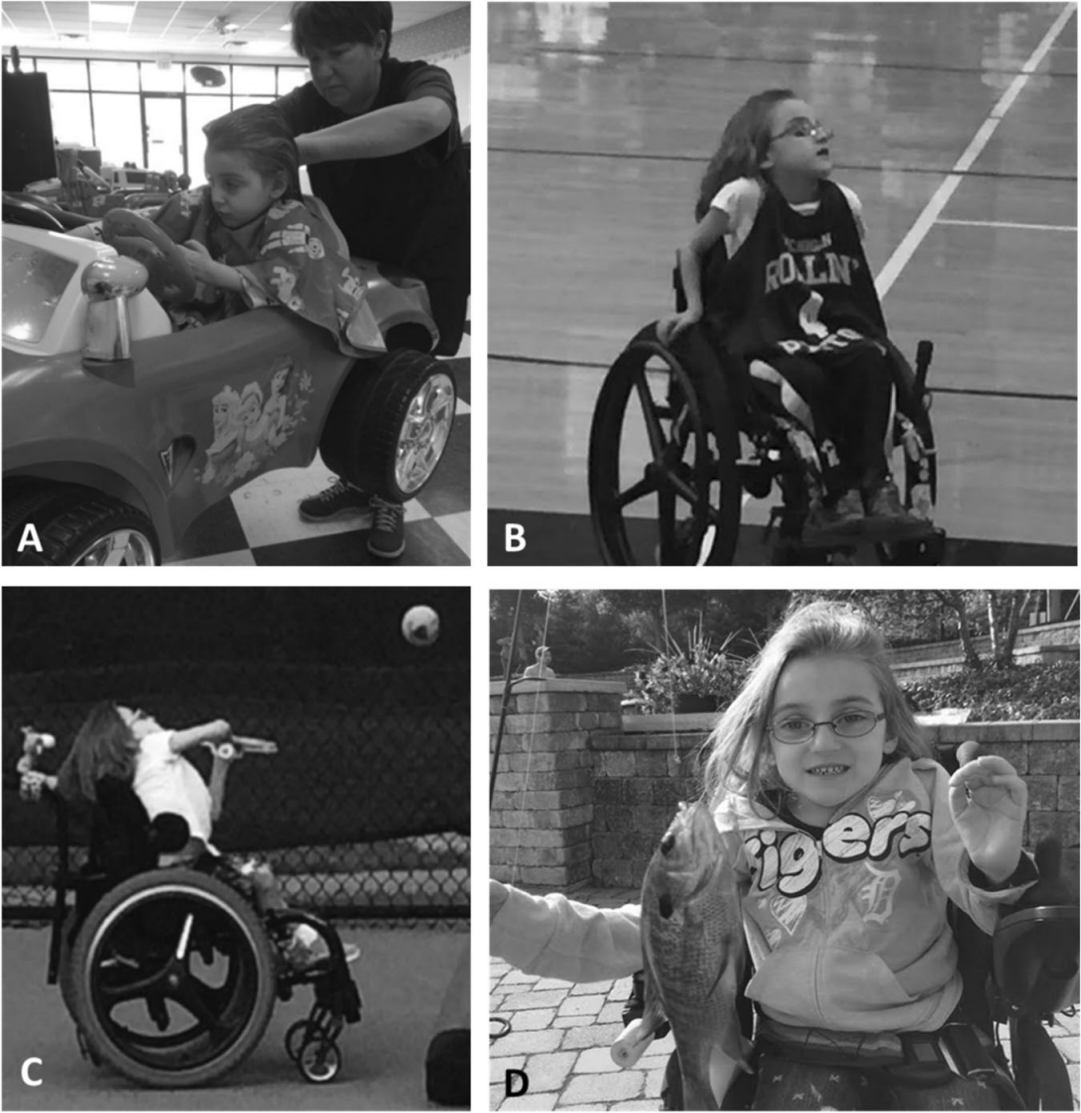
Improved quality of life and participation. **a** For the first time since injury, child was able to sit in a Barbie car for a haircut instead of having to be held in her mother’s lap. **b**, **c** She participated in numerous sports such as basketball and tennis from manual wheelchair. **d** She enjoyed fishing with her parents in the community.

At the time of discharge from AB-LT (Table 1), the child actively assisted with bilateral knee extension on command 75% of the time in supported squat position. There was a change from initial hyporeflexive response to hyperreflexive response of her left leg and from absent to hyporeflexive response on right leg. The Segmental Assessment of Trunk Control (SATCo) [21, 22], a standardized measure to assess trunk control without compensation, was first conducted during AB-LT session 23. Initially, she demonstrated appropriate kinematics during testing with arms supported on table, score of 2/20 (Head Control). She continued to improve trunk control across reassessments (Table 2). By discharge, she was able to maintain appropriate alignment of her previously kyphotic trunk position with support decreased to low ribs, 12/20 SATCo (Lower Thoracic Control), prop ring-sitting for 60 s, and was able to sit with one arm support, initiating trunk rotation to interact with her environment. In less kyphotic, upright sitting, the child’s breathing appeared automatic, unnoticeable, and void of chest raising or accessory muscle activity. She had also begun to lift her arms during short-sitting and could bimanually stack blocks without support from a ring-sitting position as well as rotate to pick up a bucket beside her with both hands and place it on her other side. Her pelvis was only slightly tilted posteriorly, and her trunk held upright with a flat back (Fig. 5a initial, 5b post-AB-LT). Improved trunk control allowed her to use her arms and hands to play. Therapists observed the mother bringing her child to therapy holding her with one arm while her child sat upright on her hip. Using this new ability to hold her upright, the child now could sit independently while having a haircut instead of her mother holding her (Fig. 6a). By discharge the child could also independently roll, come to long-sit, drag crawl, and scoot backwards in prone. Dependent wheeled mobility changed from full recline in the Kid Kart to less reclined position and the headrest was removed. While initially fully dependent, she was discharged home able to propel and steer a manual wheelchair with pelvic positioning device at a household level (Figs. 3a, b and 6b, c). She was discharged home in a custom manual wheelchair with pelvic positioning device only.

**Table 1:** Comparison of medical status and function prior to AB-LT and during/post AB-LT.

Comparison of outcomes: prior to activity-based locomotor training (AB-LT) and during/post-AB-LT		
	Prior to AB-LT	During and post-AB-LT
Duration	2.5 years	8 months
Mobility status	Dependent—-power chair/kid kart	Independent—-manual wheelchair
Functional skills	Unable to roll, sit, come to sit, stand or walk	Able to independently roll, come to sit, and sit while using arms
Medical status	Pneumonia ×2	No pneumonia (up to 3 years post AB-LT)
	Hospitalized ×2	Hospitalization ×1 (Atelectasis; 3 months post initiation of AB-LT)
	Daily use of percussion vest	No required use of percussion vest
Number of treatment sessions	~350 sessions	144 sessions

*AB-LT* activity-based locomotor training.

**Table 2:** Trunk control: progression of outcomes from initial evaluation through discharge during activity-based locomotor training (AB-LT).

Trunk control and sitting progression of outcomes			
Months of AB-LT	Session number	Outcome measure	
		SATCo	Timed sit
0	0	NT	Incapable of sitting with or without arm support
1	23	2/20	Requires arm support to sit
2	42	8/20	Requires support to sit
4	70	8/20	Requires support to sit
6	98	11/20	Starts to lift one arm during unsupported sitting
8	144	12/20	Able to sit unsupported for 30 s

Segmental Assessment of Trunk Control (SATCo) incrementally measures uncompensated trunk control with a maximum score of 20/20 representing ability to sit without external manual support while maintaining appropriate kinematics/alignment without compensation during testing of static, active, and reactive control. Timed sit measures sitting endurance without regard to alignment or kinematics.

**Fig. 7:**
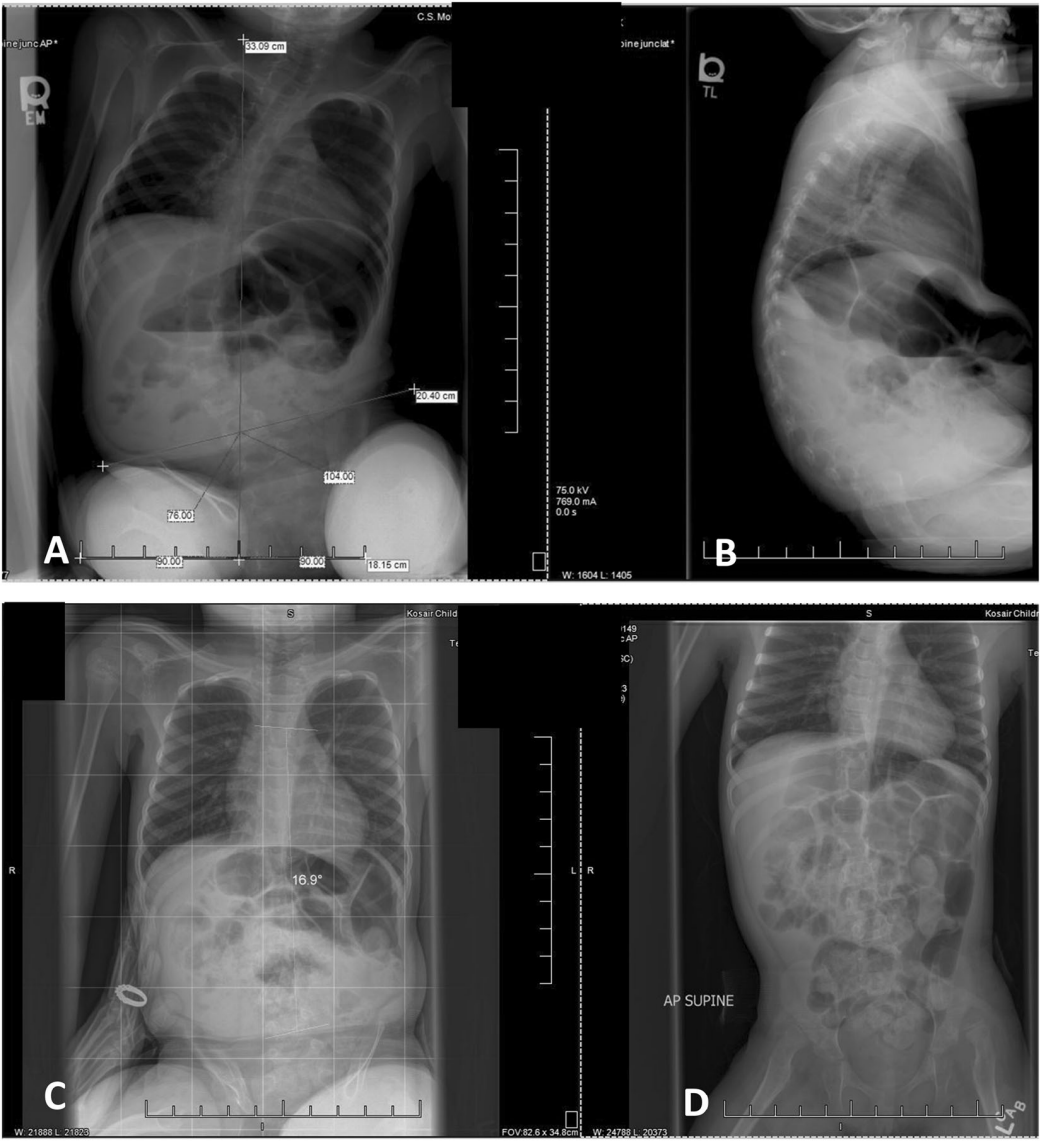
Spine x-rays diagnostic for scoliosis. Spine X-rays at age 6 years 1 month (**a**, **b**) and 6 years 5 months (**c**, **d**). At 6 years 1 month when imaged in sitting without request for active upright posture, she demonstrated a pelvic tilt of 14° and a spinal curve of 57° (**a**) as well as kyphotic posture (**b**). Four months later she was imaged again, this time she was verbally coached to “sit up tall” and use her arms to assist. She demonstrated a 16.9° spinal curve (**c**), the curve was also assessed in supine without external support (**d**) also resulting in a 17° curve.

At discharge following AB-LT, the mother described the new capacity of her daughter and the effect on caregiver burden and her child’s abilities (Fig. 3a, b). Upon return to the home and community, the child continued to be very active, expanding her participation in various sports (Fig. 6b–d). As she aged, there was a positional curve of her spine; however, the degree of the curve was dependent upon passive (Fig. 7a, b) or active sitting, as well as arm support (Fig. 7c), or whether positioned supine (Fig. 7d). Pulmonary function testing [23–26] 3–5 years post-initial AB-LT at 6–8 years of age revealed normal capacity [5, 6]. These results suggest the stability and durability of improved respiratory function years following completion of an intense program of AB-LT.

## Discussion

At 3 months of age, paralysis and immobility imposed a sedentary life on this child. Her medical history, from 3 months to 2 years 11 months, was consistent with anticipated outcomes for children injured under age 5 [1]. She had received ~350 sessions of OT/PT with no functional gains but limited self-power mobility. During and following her participation in 144 AB-LT sessions, however, this child with chronic SCI, severely compromised respiratory function, and dependence upon caregivers for all self-care and mobility, demonstrated multiple areas of improvement (Table 2). Gains occurred in trunk control, respiratory function, and mobility. The child was ultimately fully participating in everyday activities from play, to setting a table, to sports. We posit improved neuromuscular capacity was instrumental in her exploring and participating in her world (see Fig. 6). We believe her achievements in self-mobility not only benefited her physical well-being but also her development [27–31]. This link, between motor exploration and spatial aspects of development, point healthcare providers to a potential further advantage of enhancing a child’s mobility by also enhancing cognition and development.

This case report highlights the potential positive impact of AB-LT in a child with chronic, severe SCI. Other ABTs that target activation of the neuromuscular system below the lesion may also be considered and may be potentially beneficial as a recovery-based therapy, e.g., neuromuscular electrical stimulation. The mother highlighted respiratory health as a primary benefit of AB-LT, and it may have led to a reduction in healthcare utilization and hospitalization. Collectively, improved function and health appear to have enhanced the quality of life of the child and her caregivers. Whether earlier intervention with AB-LT in infants and toddlers with SCI, as young as the typical age for upright weight-bearing and steps, i.e., 9–17 months, may accelerate such improvements or reduce the incidence of secondary health and medical complications is unknown. As a case report, our intent is to disseminate information regarding this child’s unique clinical outcome and in the context of the associated clinical experience to other healthcare professionals that may treat children with SCI. A child with a chronic and severe SCI, based on level of injury, overall dependence, and history of respiratory complications, she was not expected to demonstrate significant change in physical capacity after nearly 3 years of rehabilitation intervention and a static clinical picture. This case report does not provide definitive cause–effect between the intervention and outcomes including quality of life, but raises the question concerning such possibility and potential given her medical and functional history, the targeted therapy and timely association of the outcomes in a child with a chronic SCI. Further research and reporting is warranted in children with historically such poor health and little known recovery after chronic SCI to address this possibility.

## Supplementary information

Supplemental Video 1
